# Dye-Sensitized Solar Cells with Electrospun Nanofiber Mat-Based Counter Electrodes

**DOI:** 10.3390/ma11091604

**Published:** 2018-09-04

**Authors:** Irén Juhász Junger, Daria Wehlage, Robin Böttjer, Timo Grothe, László Juhász, Carsten Grassmann, Tomasz Blachowicz, Andrea Ehrmann

**Affiliations:** 1 Faculty of Engineering and Mathematics, Bielefeld University of Applied Sciences, 33619 Bielefeld, Germany; iren.juhas_junger@fh-bielefeld.de (I.J.J.); daria.wehlage@fh-bielefeld.de (D.W.); robin.boettjer@fh-bielefeld.de (R.B.); timo.grothe@fh-bielefeld.de (T.G.); 2 Faculty of Electrical Engineering, Media Technology and Computer Science, Deggendorf Institute of Technology, 94469 Deggendorf, Germany; laszlo.juhasz@th-deg.de; 3 Faculty of Textile and Clothing Technology, Niederrhein University of Applied Sciences, 41065 Mönchengladbach, Germany; carsten.grassmann@hsnr.de; 4 Institute of Physics—Center for Science and Education, Silesian University of Technology, 44-100 Gliwice, Poland; blachowicz@posls.pl

**Keywords:** dye-sensitized solar cell (DSSC), polyacrylonitrile (PAN), nanofiber mat, electrospinning, PEDOT:PSS

## Abstract

Textile-based dye-sensitized solar cells (DSSCs) can be created by building the necessary layers on a textile fabric or around fibers which are afterwards used to prepare a textile layer, typically by weaving. Another approach is using electrospun nanofiber mats as one or more layers. In this work, electrospun polyacrylonitrile (PAN) nanofiber mats coated by a conductive polymer poly(3,4-ethylenedioxythiopene) polystyrene sulfonate (PEDOT:PSS) were used to produce the counter electrodes for half-textile DSSCs. The obtained efficiencies were comparable with the efficiencies of pure glass-based DSSCs and significantly higher than the efficiencies of DSSCs with cotton based counter electrodes. The efficiency could be further increased by increasing the number of PEDOT:PSS layers on the counter electrode. Additionally, the effect of the post treatment of the conductive layers by HCl, acetic acid, or dimethyl sulfoxide (DMSO) on the DSSC efficiencies was investigated. Only the treatment by HCl resulted in a slight improvement of the energy-conversion efficiency.

## 1. Introduction

Dye-sensitized solar cells (DSSCs) are being investigated intensively since their development in 1991 [1]. In the last years, typical conversion efficiencies in the lab reached about 11–29% [2,3,4,5], with commercially available cells showing efficiencies around 2–3%. Typically, ruthenium-based dyes are used as sensitizers although they are expensive and in most cases toxic [6,7]. Recently, DSSCs sensitized by porphyrin dyes were investigated [2,7,8,9] and even higher efficiencies than by the cells with Ru-based dyes were achieved [2]. Cells with metal-free organic dyes have slightly lower efficiencies, but since containing no noble metals, they are less cost intensive [7,10]. Even metal-complexes have been examined as sensitizers for DSSCs [11,12].

Natural dyes, on the other hand, are inexpensive and non-toxic, but show significantly reduced efficiencies [13,14,15,16,17,18]. This, however, is less important if large areas can be used—which is the case, e.g., for textile architecture and other outdoor textiles. Natural dyes are also used in this article.

Due to the possibility to work with such inexpensive, non-toxic materials outside a cleanroom, several approaches were used to create textile-based DSSCs [19,20,21,22,23]. Amongst them, however, only few concentrate on nanofiber mats produced by electrospinning [24,25]. This method allows for creation of nanofiber mats from diverse polymers or polymer blends, amongst them several biopolymers [26,27,28,29], man-made polymers [30,31], or blends with inorganic compounds [32,33,34]. Such electrospun nanofiber mats are used for a broad range of applications, from wastewater filters [35] to catalyzers [36] to medical wound dressings or cell growth substrates [37,38,39,40] to precursors for carbon nanofibers which may be included in polymers to enhance their mechanical properties [41,42,43].

However, only few reports can be found about integration of electrospun nanofiber mats in DSSCs. Such nanofiber mats were, e.g., used to create quasi-solid electrolytes for the integration in DSSCs [44,45]. Electrospun TiO_2_ nanofibers were used to create the photo-anode [46,47]. Electrospun carbon nanofibers were applied with different coatings or blends as counter electrodes [48,49].

The simple approach of coating a non-conductive nanofiber mat—which can unambiguously be produced by electrospinning, opposite to conductive nanofibers which need a two-step procedure—by poly (3,4-ethylenedioxythiopene) polystyrene sulfonate (PEDOT:PSS), a conductive polymer, to combine the large surface–volume ratio of the nanofiber mat with the good conductivity of PEDOT:PSS, was nevertheless not yet found in the scientific literature. This article thus aims at showing in a first-principle study that PEDOT:PSS-coated nanofiber mats can be used as textile counter electrodes, investigating half-textile DSSCs in which the counter electrodes are replaced by textile substrates with conductive coatings (PEDOT:PSS), while the working electrodes are still made from glass and are prepared using a natural dye. DSSCs with counter electrodes prepared on PAN nanofiber mats and PAN nano-membranes are compared to reference cells with counter electrodes prepared on fluorine-doped tin oxide (FTO)-coated glass and on cotton as a macroscopic textile fabric. The effects of different numbers of PEDOT:PSS coating layers as well as of after-treatments of the PEDOT:PSS by dimethyl sulfoxide (DMSO), HCl, or acetic acid which is reported in the literature [50] were investigated to increase the conductivity of the counter electrode. The resulting efficiencies are similar to or even significantly higher than those achieved with common glass-based approaches.

## 2. Materials and Methods

### 2.1. Preparation of the Counter Electrodes (Textile Half-Cell)

Nanofiber mats were prepared using the electrospinning machine “Nanospider Lab” (Elmarco, Czech Republic), a needleless electrospinning machine based on the wire technology. For the spinning solution, 16% PAN were dissolved in DMSO (min 99.9%, from S3 Chemicals, Bad Oeynhausen, Germany). Spinning was performed for 1 h 25 min with the following spinning parameters: voltage 65 kV, electrode-substrate distance 240 mm, nozzle diameter 0.8 mm, carriage speed 50 mm/s, substrate speed 0 mm/min, relative humidity 32–33%, and temperature 22.0 °C. The resulting nanofiber mat was 0.105 mm thick with an areal weight of 21.08 g/m^2^.

The PAN membranes were produced by treating the electrospun nanofiber mat with 11.25 µL DMSO per cm^2^ and drying at 50 °C on a hot plate. The DMSO treatment dissolves the PAN transforming the original nanofiber mats into a membrane.

For comparison, a cotton fabric (thickness 0.19 mm, areal weight 84.49 g/m^2^) and FTO-coated glass plates (purchased from Man Solar, Petten, The Netherlands) were used. 

The nanofiber mats and nano-membranes as well as the cotton fabric were placed on microscopy slides and coated by doctor blading with 11.25 µL PEDOT:PSS per cm^2^ area (1 to 5 times) to make them conductive. For this, CLEVIOS™ S V 4 (sheet resistance 500 Ohm) and Orgacon™ S305 (sheet resistance 200 Ohm) were used. The sheet resistances differ strongly, enabling the investigation of the influence of this parameter. After each layer the textiles were heated to 110 °C together with the microscopy slides for 50 min in an oven. The microscopy slides served as support for the textile electrodes.

To increase the conductivity, some of the samples with a single PEDOT:PSS layer were treated by 11.25 µL/cm^2^ DMSO, 0.2 mol/L HCl, or 80% (vol.) acetic acid and heated for 10 min to 110 °C, 140 °C, or 160 °C, respectively, as proposed in Ref. [50]. After cooling to room temperature, the DMSO, the HCl and the acetic acid were removed by dipping the samples into distilled water. The textile and glass counter electrodes were coated with graphite spray (Graphit 33 by Kontakt Chemie), spraying for ca. 1 s from a distance of 25–30 cm, and heated to 90 °C for 60 min in an oven.

### 2.2. Preparation of the Working Electrodes (Glass Half-Cell) and Cell Assembly

The working electrodes were prepared using TiO_2_-coated FTO glasses (Man Solar). For the dye, 25 g forest fruit tea (Mayfair, Wilken Tee GmbH, Fulda, Germany) were ground, mixed with 300 mL distilled water, and stirred with 200 min^−1^ at 40 °C for 30 min. Afterwards, the solid contents were filtered. The TiO_2_ coated plates were inserted into the dye solution at room temperature for 2 days to avoid potential irregularities due to unequal dyeing times. The excess dye was washed off with distilled water, and the plates were dried at the air.

Both electrodes were pressed together and fixed with adhesive tape. A iodine/triiodide-based electrolyte (electrolyte type 016 purchased from Man Solar) was dropped on the gap between both electrodes and left to spread by capillary force. The cells were not sealed. The measurements of the I-U characteristics were taken with unmasked solar cells with an active area of 6 cm^2^.

### 2.3. Measurements

Confocal laser scanning microscope (CLSM) images were taken with a VK-9000 (Keyence, Neu-Isenburg, Germany) with a nominal magnification of 2000×. All scale bars have dimensions of 10 µm.

Current-voltage (I-U) curves were measured with a source meter unit 2450 SourceMeter (Keithley Instruments, Solon, OH, USA) in the voltage range from 0.5 to 0 V. A halogen lamp with a color temperature of 3000 K was used for these investigations. The light intensity was measured by a solarimeter KIMO SL-200 and set to 1000 W/m^2^ on the DSSC surface.

The spectral analysis and parameter identification of the linearized DSSC model was performed as described in detail in Ref. [51].

Three DSSCs are prepared with each type of counter electrodes and their current-voltage (I-U) characteristics were averaged.

## 3. Results and Discussion

### 3.1. Effect of the Textile Substrate

First, the effect of the textile substrate of the counter electrode on the photovoltaic characteristics of the DSSCs is investigated. For that purpose, the counter electrodes for the DSSCs are prepared from a PAN nanofiber mat, PAN nano-membrane and cotton, coated by a single layer of PEDOT:PSS (S305 or S V 4). For comparison, conventional cells with counter electrodes made from FTO glass were also prepared.

Table 1 shows the open-circuit voltages, short-circuit currents, fill factors, and efficiencies of these cells. The energy-conversion efficiency η of the cells is calculated from the I-U characteristics averaged for the three cells with the same counter electrode as $\eta /\%=100\cdot FF\cdot U_{OC}\cdot I_{SC}/\left(A\cdot I_{0}\right)$, where $U_{OC}$ is the open-circuit voltage, $I_{SC}$ is the short-circuit current, A is the cell area of 6 cm^2^, $I_{0}$ is the incident irradiation, and FF is the fill factor defined as $FF=U_{m}\cdot I_{m}/\left(U_{OC}\cdot I_{SC}\right).$ $U_{m}$ and $I_{m}$ are the photovoltage and photocurrent of the maximum power of the DSSC.

**Figure 1:**
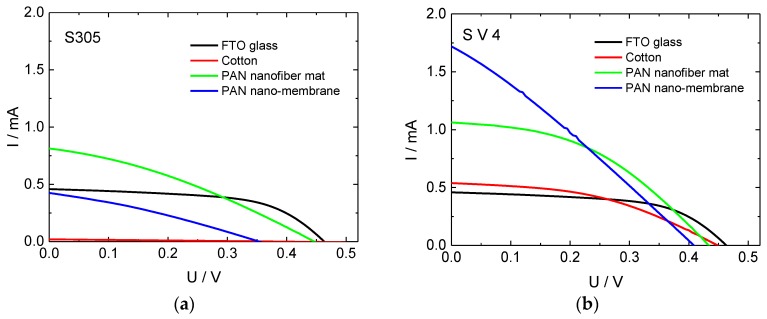
Current-voltage curves of dye-sensitized solar cells (DSSCs), prepared with different counter electrodes: (**a**) S305 as conductive coating; (**b**) S V 4 as conductive coating.

In Figure 1, the corresponding current-voltage characteristics are depicted. The pure FTO glass cells show relatively rectangular I-U curves, verifying a higher fill factor than the other cells shown here. While the cotton cells prepared with S305 have currents near zero, the ones produced with S V 4 have even slightly higher short-circuit currents than the glass cells, but a reduced fill factor.

Comparing the results from both sorts of PEDOT:PSS used in this investigation, it can be recognized that the cells prepared with S V 4 show slightly higher currents although this PEDOT:PSS has a higher sheet resistance. This shows that the conductivity is not the only relevant factor.

Especially the nanofiber mat and the nano-membrane prepared with S V 4, although both with reduced fill factors, have significantly higher short circuit currents than the cells prepared on glass. For S305, the nanofiber mat still shows a higher short-circuit current than the FTO glass.

**Figure 2:**
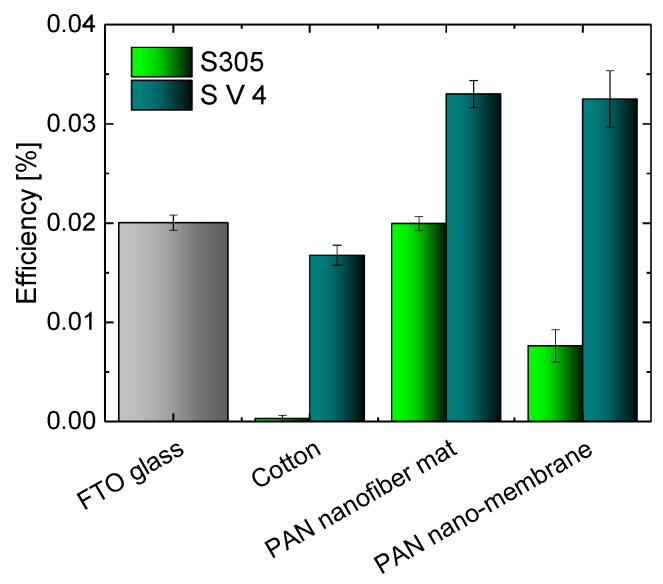
Efficiencies of DSSCs prepared with different textile-based counter electrodes and FTO glass as a reference.

Figure 2 depicts a comparison of the efficiencies of DSSCs with counter electrodes built on different textile substrate with the efficiency of pure glass cell. As usual for DSSCs prepared from non-toxic natural substances, the efficiencies are relatively low. However, the best textile-based cells reach the efficiencies which are typically gained with glass-based cells using otherwise similar materials [15,17] and even clearly outperform the reference glass cell prepared with identical materials by more than 50%, underlining that nanofiber mats and nano-membranes coated with PEDOT:PSS are indeed a well-suited alternative for FTO glasses which are typically used for low-cost, non-toxic DSSCs. It should be mentioned that while with the lower-conductive PEDOT:PSS S V 4, nanofiber mat and nano-membrane show very similar efficiencies, the nano-membranes coated with S305 have clearly smaller efficiencies than the corresponding nanofiber mat. On the other hand, the efficiencies gained with cotton are lower than those obtained with pure glass cells, indicating that such macroscopic textile fabrics are not the ideal choice for the creation of textile-based solar cells. Since the measurements of the I-U characteristics were performed with unmasked DSSCs, the efficiencies of all cells may be slightly overestimated. Snaith et al. reported a decrease of the efficiency by about 30% when measured with masked cells compared to the measurements with unmasked cells [52]. The reason for the overestimation of the efficiency in measurements without mask is that in this case, not only the light falling on the active area can enter the cell, but the light falling on the glass boarder surrounding the active area or on the edges of the cell may also be trapped by the glass and enter the cell, contributing to the energy conversion. Our DSSCs have glass parts bordering the active area only at one edge. In half-textile DSSCs, extra light can enter the cells through these small glass areas and through the edges of the working electrodes, while the counter electrodes are not transparent. Therefore, we believe that in our case, the grade of the efficiency overestimation is reduced compared to that reported by Snaith et al.

**Figure 3:**
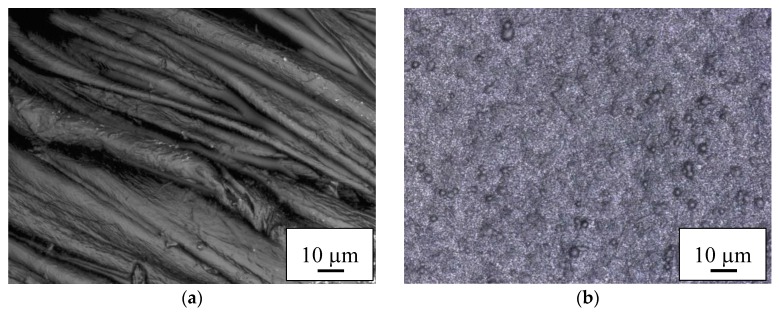

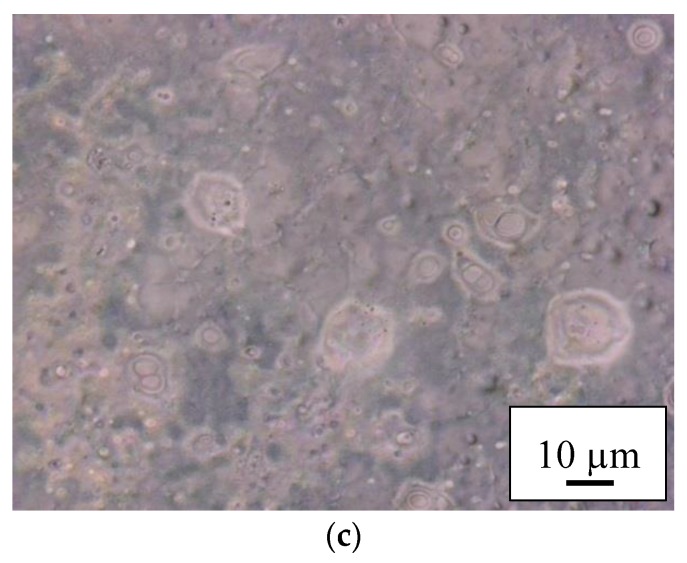
Confocal laser scanning microscope (CLSM) images of the textile-based counter electrodes prepared with S305 on (**a**) cotton; (**b**) PAN nanofiber mat; and (**c**) PAN nano-membrane taken with nominal magnification 2000×. The length of the scale bars is 10 µm in all subfigures.

**Figure 4:**
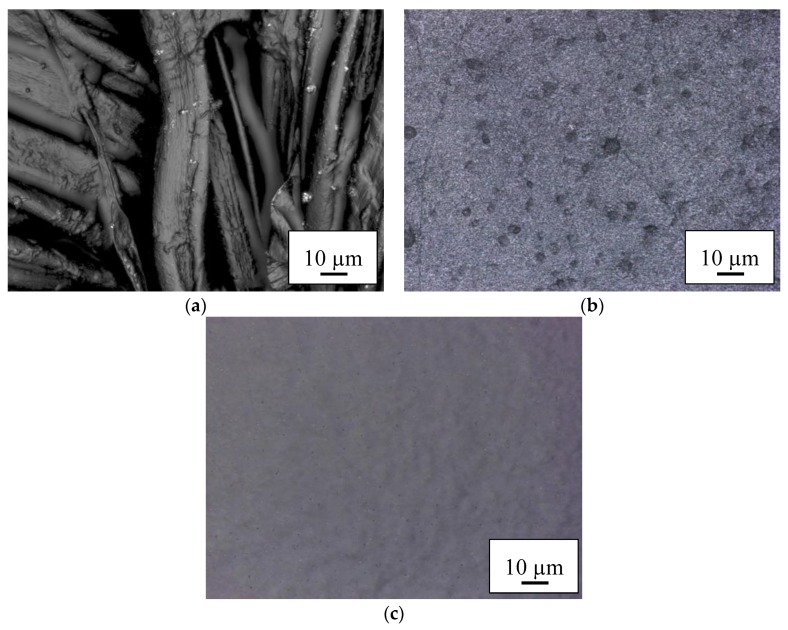
Confocal laser scanning microscope (CLSM) images of the textile-based counter electrodes prepared with S V 4 on (**a**) cotton; (**b**) PAN nanofiber mat; and (**c**) PAN nano-membrane taken with nominal magnification 2000×.

The results of optical examination of the PEDOT:PSS-coated nanofiber mats and membranes are depicted in Figure 3 and Figure 4. In both cases, coating cotton with PEDOT:PSS (Figure 3a and Figure 4a) shows a thin coating on the fibers, with some interconnections due to the coating, as expected. The macroscopic fibers nevertheless impede creation of a closed conductive layer.

Coating the PAN nanofiber mats with PEDOT:PSS (Figure 3b and Figure 4b) reveals the typical nanofiber mat structure with some small agglomerations which are typical for PEDOT:PSS. As expected, the membranes (Figure 3c and Figure 4c) do not show any fibrous regions.

The sealing of the half-textile DSSCs is still an unsolved problem since the electrolyte evaporates not only on the edges of the cells, but through the whole surface of the textile electrode. This leads to a loss of efficiency in a relatively short period of time.

### 3.2. Effect of the Number of Conductive Layers

As we have seen in the previous subsection, the efficiency of some textile-based DSSCs reaches the efficiency of pure glass cells, but is still low. It could be increased by decreasing the inner resistance of the cell by enhancing the conductivity of the PEDOT:PSS coating on the counter electrode. One of the possibilities to reach this goal is applying more than one PEDOT:PSS layer, as proposed in the literature [53,54].

**Figure 5:**
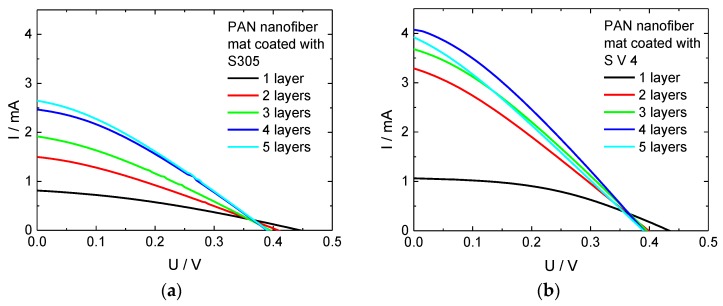
Current-voltage curves of DSSCs, prepared with different counter electrodes: (**a**) S305 as conductive coating; (**b**) S V 4 as conductive coating.

As a substrate for the counter electrodes, PAN nanofiber mats were used due to the above described findings that they are less sensitive to the choice of the PEDOT:PSS than nano-membranes, can be prepared in one production step (cf. Section 2.1) and show higher fill factors. The nanofiber mats were coated up to five times with PEDOT:PSS. The results are depicted in Figure 5. Coating the samples with PEDOT:PSS twice results in a significant increase of the short circuit currents for both PEDOT:PSS versions. This can be explained by monitoring the coating process carefully: The first PEDOT:PSS layer flows into the nanofiber mat where it is partly disconnected by the non-conductive PAN nanofibers. The second layer is placed on top of the first one and can form a more continuous conductive layer, in this way strongly increasing the current transport along the nanofiber mat.

Adding more PEDOT:PSS layers, the short circuit currents continuously increase (for S305) or increase until a maximum is reached for four layers (for S V 4), respectively. The open circuit voltages decrease slightly from one layer to two layers and stay constant afterwards.

**Figure 6:**
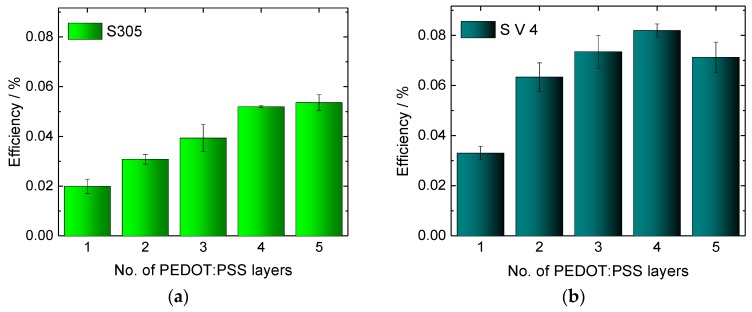
Efficiencies, obtained with PAN nanofibers mats and different numbers of PEDOT:PSS layers. (**a**) S305 as conductive coating; (**b**) S V 4 as conductive coating.

Figure 6 depicts the corresponding efficiencies. While the value of the glass cells prepared as benchmark (0.02%) is even reached for one layer of the PEDOT:PSS S305, adding further layers clearly increases this value, until saturation is approached after 4 layers for both PEDOT:PSS versions, indicating that the cost–benefit ratio may be ideal for this number of layers.

For S V 4, the maximum efficiency of 0.08%, i.e., four times the value of the glass reference, is reached for four layers. Unexpectedly, the efficiency of the cells with five layers of S V 4 is slightly decreased compared to the efficiency of cells with four layers of S V 4. A similar observation is made for the current, i.e., the I-U curve of the cells with five layers of S V 4 is lower than the I-U curve of cells with four layers. The reason may be an increased inner resistance of the cells with five S V 4 layers in comparison to cells with four layers. To test this assumption, AC measurements followed by spectral analysis were performed [51]. According to the obtained results (Table 2) there is a clear correlation noticeable between the number of layers and the linearized resistance of the textile-based DSSC. With increasing the number of layers up to four layers, the linearized dynamic resistance decreases. The DSSC with five layers of S V 4 has, however, an increased resistance related to the four-layer one. This justifies our assumption and explains the drop of the efficiency of five-layer cell. The difference between both systems under investigation may be attributed to S V 4 necessitating a smaller number of layers to reach the ideal combination of high conductivity and low thickness, both of which will reduce the inner resistance of the cells.

### 3.3. Effect of Chemical Post-Treatment of the Conductive Layer

In the previous subsection, it was shown that the conductivity of PEDOT:PSS and therewith the efficiency of the cells can be increased by coating more than one conductive layer on the counter electrode. However, increasing the number of coating layers is cost consuming and results in losing the textile nanostructure after several coating layers. Therefore, an alternative solution should be found.

**Figure 7:**
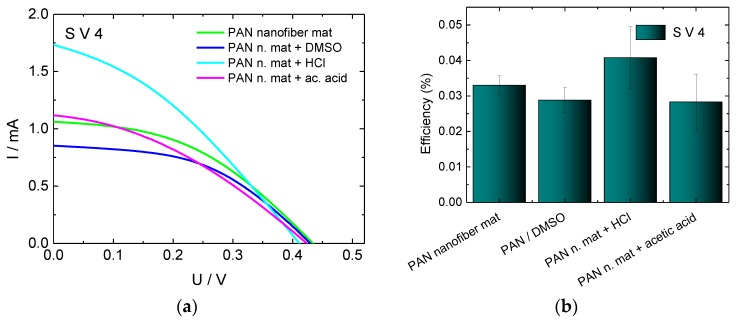
(**a**) Current-voltage characteristics and (**b**) efficiencies of DSSCs with counter electrodes prepared from PAN nanofiber mat coated by a single layer of S V 4, pure and with a post treatment by DMSO, HCl, or acetic acid.

In Ref. [55] and the references therein, the possibility to enhance the conductivity of PEDOT:PSS layers on a glass substrate by more than two orders of magnitude by adding polar organic molecules such as DMSO, ethylene-glycol, diethylene glycol, or sorbitol to the aqueous solution of PEDOT:PSS was reported. According to Refs. [50,56], a chemical post-treatment of the PEDOT:PSS layer by inorganic acids (hydrochloric acid, sulfurous acids), organic acids (acetic acid, propionic acid etc.), and inorganic solvents (DMSO, ethylene glycol, etc.) results in an enhancement of the conductivity by a factor greater than 1000. Inspired by these encouraging results, the nanofiber mats coated with a single layer of S V 4 (the other PEDOT:PSS, S305, was omitted since the cells built with it yielded lower efficiencies in previous investigations) were treated with DMSO, HCl or acetic acid, as described in detail in Section 2.1, and the resulting counter electrodes were used to prepare DSSCs. The I-U characteristics and the efficiencies of the obtained cells are depicted in Figure 7.

While a clear change of the short-circuit current could be observed, the open circuit voltage remained nearly the same as for the cells without chemical treatment. Since a significant enhancement of the conductivity of PEDOT:PSS layer through the chemical treatment was reported in the literature, we expected also a significant increase of the efficiency of DSSCs compared to cells with untreated conductive layer. However, only the cells treated with HCl show a slight efficiency enhancement. This finding can be attributed to the much lower concentration of the HCl used here in comparison to the experiment described in the literature. In Ref. [50] it was shown that the conductivity increase depends on the concentration of the used HCl and reaches its maximum at a concentration of 9.6 mol/L. Since highly concentrated HCl could damage the nanofiber mat, the optimal concentration has to be found in future tests.

The DSSCs treated with DMSO or with acetic acid showed a slight decrease of the efficiency instead of an efficiency increase. It is known that DMSO dissolves PAN. Through the DMSO treatment of the PEDOT:PSS layer on a PAN nanofiber mat, the PAN mat is dissolved leading to mixing of PEDOT:PSS with PAN. The nanofiber structure is lost, and a membrane is formed. By forming poorly connected PEDOT:PSS islands within the isolating PAN membrane, the conductivity of the PEDOT:PSS layer is decreased. This results in a higher inner resistance and decreased efficiency of corresponding DSSCs. We assume that the efficiency drop of acetic acid treated cells may have the same reason.

## 4. Conclusions

In this paper, half-textile DSSCs with textile-based counter electrodes coated by a conducting polymer and working electrodes built on TiO_2_ coated FTO glass dyed with a natural dye were investigated. The conductive coating was performed by two types of PEDOT:PSS: by CLEVIOS™ S V 4 and by Orgacon™ S305.

Coating of non-conductive electrospun PAN nanofiber mats with conductive PEDOT:PSS has been shown to result in high-quality counter-electrodes for half-textile DSSCs, with efficiencies comparable to those gained with pure FTO-glass cells even for the lower-conductive PEDOT:PSS used in this investigation and clearly superior to those of PEDOT:PSS coated cotton. The efficiency was dependent on the number of coating layers. Using 4 layers of S V 4, the efficiency could be increased by a factor of 4, as compared to the FTO-glass reference cells. 

Additionally, a post-treatment of the conducting layer with DMSO, acetic acid or HCl was investigated. Only the treatment with HCl led to a slight increase of the efficiency, whereas the efficiency of cells treated with DMSO and acetic acid was slightly decreased compared to the untreated cells. This was ascribed to dissolving or damaging the nanofiber mat by treatment with both latter materials and forming of an only partially conducting layer.

Generally, this study has shown that electrospun nanofiber mats are a good alternative to common glass-based electrodes, offering a method to create a nanostructured electrode with a simple, textile-based technology. Aiming at increasing the efficiency, in the future the influence of the nanofiber mat morphology, as defined by the spinning and solution parameters, will be studied. The HCl-treatment will be optimized and performed on coatings with more than one conductive layer. The possibilities of sealing the half-textile cells will be investigated.

## Figures and Tables

**Table 1:** Open-circuit voltage $U_{OC}$, short-circuit current $I_{SC}$, fill factor *FF*, and efficiency η for cells prepared with different textile fabrics and sorts of PEDOT:PSS in comparison to FTO glass cells.

PEDOT:PSS	Counter Electrode	U_OC_/V	*I_SC_*/mA	FF	*η*/%
	FTO glass	0.462	0.46	0.57	0.020
S305	Cotton	0.387	0.02	0.24	3 × 10^−4^
	PAN nanof. mat	0.448	0.81	0.33	0.020
	PAN membrane	0.354	0.42	0.31	0.008
S V 4	Cotton	0.449	0.54	0.41	0.017
	PAN nanof. mat	0.434	1.06	0.43	0.033
	PAN membrane	0.408	1.72	0.28	0.033

PEDOT:PSS, poly(3,4-ethylenedioxythiopene) polystyrene sulfonate; FTO, fluorine-doped tin oxide; PAN, polyacrylonitrile.

**Table 2:** Resistance R_1_ of cells prepared with S V 4, calculated from pole-zero transfer function (TF) estimation, and resistance R_2_, calculated by the equivalent-circuit TF estimation [51].

Cell Type	R_1_/Ω	R_2_/Ω
Single layer	575.91	755.53
Two layers	528.58	529.60
Three layers	279.14	288.14
Four layers	130.47	165.70
Five layers	242.43	253.66
